# To the Editors of the Medical and Physical Journal

**Published:** 1807-06

**Authors:** S. Sawrey

**Affiliations:** Chancery Lane


### *To the Editors of the Medical and Physical Journal.*

Gentlemen,

In your Review of my pamphlet on the newly-discovered membrane in the human eye, I observe that an error of the press has escaped your notice. I do not believe that another fluid may be interposed between that membrane and the transparent cornea. My opinion is, that the membrane being interposed between the cornea and the aqueous humour, prevents that fluid from coming into contact with the cornea, which would otherwise penetrate its porous texture, and render it thick and opaque; — so important appears to be the use of this membrane.

I take this opportunity of mentioning in your valuable publication, an experiment that I have since made, which I think proves the truth of the above conclusion. I procured the eyes of a horse, the cornea of which were perfectly transparent. After dividing the sclerotic coat transversely, I took out the remaing humours along with the choroides and iris, and in one of them the membrane which lines the cornea. I then filled them with water, suspending them with small pieces of thread. After three days, the cornea of the one which had the membrane left in, remained perfectly transparent; nor could I perceive it to be in the smallest degree thickened. The cornea of the other was quite white, and swollen to three or four times its usual thickness.

I am, &c.

*Chancery Lane, March* 7, 1807. S. SAWREY.

---

